# SIESTA Project: Svalbard summer 2025 expedition report

**DOI:** 10.12688/openreseurope.21683.1

**Published:** 2026-01-12

**Authors:** Rey Mourot, Sibylle Lebert, Eloi Martinez-Rabert, Aude Barani, Gérald Grégori, Sandra Nunige, Aurélie Dufour, Sophie Guasco, Catherine Larose, James A. Bradley

**Affiliations:** 1 Aix Marseille Université, Université de Toulon, CNRS, IRD, MIO, Marseille, 13009, France; 2 Aix-Marseille Université, CNRS, IMM, LCB, Marseille, France; 3 IGE—Institut des Géosciences de l’Environnement, Université Grenoble Alpes, Saint Martin d’Hères, France; 4 School of Biological and Behavioural Sciences, Queen Mary University of London, London, England, UK

**Keywords:** microbial dormancy, cryosphere, glacier ecology, arctic, microbial ecology, Svalbard, Glacier ecosystems, Svalbard, Single-cell activity, BONCAT.

## Abstract

Microbial dormancy plays an important role in the persistence, dispersal, and functioning of microbial communities in moderate to extreme environments. The activity or inactivity of microbial communities also has implications for rates of biogeochemical transformations and thus elemental stocks and redox conditions. Microbial communities inhabiting glacier surface environments encounter harsh and variable environmental conditions including nutrient limitation, low temperatures, and light availability across various micro-habitats including cryoconite and the bare ice surface. The metabolic states of cells within these microhabitats and in relation to their environment is fundamental to the functioning of the ecosystem and has implications for ecosystem resilience, responses to environmental change, and biogeochemical cycling. This report describes an expedition to Brøggerhalvøya, north-west Svalbard, carried out in July 2025, within the framework of the ERC SIESTA project. A major objective of the project is to resolve microbial activity and dormancy on an individual cell basis, to characterise the adaptive and functional traits of active and dormant fractions of the native glacier microbial population, and to link microbial metabolic states to broader ecological and biogeochemical dynamics. Here we report the site characteristics, the samples collected, the analyses undertaken, and the future analyses planned. Two small valley glaciers near to Ny-Ålesund were selected for investigation during this summer campaign: Midtre Lovénbreen and Austre Brøggerbreen. The data collected in the field, combined with subsequent laboratory analyses, will provide insights into the spectrum of dormancy and activity in situ among glacier microbial communities, and the taxa and functions associated with active and inactive fractions of the communities. These findings will contribute to a deeper understanding of the impacts and role of both short- and long-term microbial dormancy in glacial environments.

## Plain language summary

Glaciers are cold and harsh environments, but they are not lifeless. Glaciers host diverse microbial communities that survive in surface ice and in small water and debris-filled holes called cryoconite. These microbes drive important processes such as nutrient cycling and carbon transformations. However, because glaciers pose challenging conditions to life, and because conditions can change quickly, many microbes survive by entering a dormant (inactive) state and “waking up” only when conditions improve. Understanding when microbes are active or dormant is crucial for predicting how glacier ecosystems function and how they will respond to climate change. Yet, we still know very little about how common dormancy is on glaciers, which microbes are active or inactive, and what roles they play in the environment. As part of the ERC-funded SIESTA project, we carried out a field expedition in July 2025 to Svalbard, working on two small valley glaciers (Midtre Lovénbreen and Austre Brøggerbreen) near the research town of Ny-Ålesund. Our goal was to investigate microbial activity and dormancy directly in natural microbial habitats on glaciers. We collected samples from different supraglacial environments and used a range of methods to label and identify active and dormant cells at the single cell level. In this report, we describe the study sites, the samples we collected, the analyses we performed in the field, some logistical considerations, and the laboratory work that will follow. The data we generate will reveal how many microbes are active or dormant, which species are involved, and what functions they carry out. This will help us better understand how dormancy shapes glacier ecosystems and influences how they respond to ongoing environmental changes.

## Introduction

Microbial dormancy - an acute and reversible reduction of metabolic activity - is widespread across ecosystems ( Jones & Lennon, 2010). It serves as a key survival mechanism that enables persistence during stress and rapid recovery after disturbance ( Lennon & Jones, 2011), and may be induced by fluctuating environmental conditions or arise stochastically as a bet-hedging strategy within populations ( Măgălie *et al.*, 2023). In glacier ecosystems, microorganisms encounter recurrent nutrient scarcity, freezing and thawing, and extreme seasonal variations in light availability, UV intensity and temperature. Dormancy is thought to underpin survival during these unfavourable periods while permitting rapid reactivation when conditions improve ( Kussell & Leibler, 2005). Because dormant cells contribute little immediately to biogeochemical cycles, the balance between active and inactive fractions strongly influences primary production, elemental cycling, and microbial community resilience ( Bradley, 2025; Shoemaker *et al.*, 2022; Shoemaker & Lennon, 2018).

Arctic glaciers are undergoing rapid transformation under climate warming, with Svalbard experiencing some of the fastest rates of change globally ( Previdi *et al.*, 2021; Rantanen *et al.*, 2022). These environmental alterations change melt dynamics, nutrient export, and microbial habitat stability ( Bradley *et al.*, 2025; Hodson *et al.*, 2008); and thus understanding how microbial dormancy modulates ecosystem responses is critical to predicting future changes to these systems. Recent methodological advances have enabled direct detection of microbial activity in situ on an individual cell basis. For example, bioorthogonal non-canonical amino acid tagging (BONCAT) ( Hatzenpichler *et al.*, 2014) can be used to fluorescently label translationally active cells, and has been applied to a range of environments including soils ( Couradeau *et al.*, 2019) and the deep ocean ( Peoples *et al.*, 2018), as well as to glacial environments to visualise active and inactive cells via microscopy ( Bradley *et al.*, 2023). However, the precise proportions, taxonomic identity, and functional capabilities of these active and dormant fractions remain unresolved. Moreover, microbial activity should be conceptualized as a continuum, from actively dividing to metabolically quiescent, rather than a binary distinction. Defining operational thresholds for dormancy, and quantifying its prevalence and ecological role, remain major challenges.

The SIESTA project aims to address these challenges by resolving microbial activity and dormancy at the single-cell level and linking these states to ecological and biogeochemical dynamics. High Arctic glaciers provide an ideal natural laboratory for this work - being pristine and extreme environments with pronounced seasonal cycles of continuous light and darkness. Svalbard, being in the fastest-warming region on Earth, also serves as a setting for investigating how microbial communities respond to rapid climate change. We therefore conducted a field expedition to Svalbard in July 2025 to perform single-cell activity assays directly in situ across a range of glacial environments.

This expedition report summarizes the fieldwork, describes the study sites and the sampling, and recounts the activities carried out both in the field and in the field laboratory. We also provide an overview of the subsequent experiments that will be conducted in the home laboratory. Our fieldwork was based in Ny-Ålesund, a small settlement in north-west Svalbard, hosting an international research community. Conducting fieldwork on High Arctic glaciers requires specific considerations, including careful planning, with attention to remote outdoor work, harsh environmental conditions, glacier safety, polar bear precautions, and logistical constraints. Precautions to mitigate risks from polar bears on Svalbard include carrying flare pistols and bolt-action rifles (used only as a last resort), which the field team are trained and certified to use. Our research project was carried out from the French–German AWIPEV Arctic Research Base ( https://www.awipev.eu), whose field support included radio communications and provision of polar bear protection equipment. Our campaign focused on two well-studied glaciers near Ny-Ålesund, Svalbard: Midtre Lovénbreen and Austre Brøggerbreen. These two glaciers are reachable on foot from Ny-Ålesund during summer, and by snowmobile or skis during winter, making them well-suited for repeated investigations across seasons. These two glaciers have been the focus of microbiological and biogeochemical research for several decades, providing a valuable legacy of environmental and microbiological data (e.g. Anesio *et al.*, 2009; Fleming *et al.*, 1997; Irvine-Fynn *et al.*, 2014). We targeted two principal supraglacial habitats on each glacier: (i) surface glacier ice and (ii) cryoconite holes. These habitats are known to host microbial communities with contrasting diversity and activity profiles ( Anesio *et al.*, 2017; Edwards *et al.*, 2011; Zeng *et al.*, 2013). Our sampling strategy was designed to capture these diverse habitats and provide a representative basis for quantifying the spectrum of microbial metabolic states on glacier surfaces. After collection, samples were transported to the field laboratory and subjected to a set of incubation treatments to distinguish the physiological state of microbial cells. These included:

- BONCAT ( Hatzenpichler *et al.*, 2014) - to identify translationally active cells by incorporating a synthetic amino acid analogue into newly synthesized proteins.
- RedoxSensor™ Green (RSG) ( Kalyuzhnaya *et al.*, 2008; Konopka *et al.*, 2011) - to detect cells with active electron transport chains associated with intracellular reductase activity.
- 5-ethynyl-2'-deoxyuridine (EdU) ( Salic & Mitchison, 2008) - to label cells undergoing DNA replication.
- Propidium monoazide (PMA) ( Nocker *et al.*, 2006) - to detect membrane-compromised cells and extracellular DNA, providing context for distinguishing dormancy from death.

Together, these assays move beyond traditional live–dead staining by targeting distinct physiological states along a spectrum, rather than considering a binary (dormant versus active) classification.

Subsequent flow cytometry (carried out at the PRECYM platform; https://precym.mio.osupytheas.fr/) analyses enable high-throughput quantification of microbial cells labelled with these various markers at the single-cell level. Flow cytometry and cell sorting allows separation of microbial populations according to each marker, for downstream genomic sequencing. This combination provides both taxonomic and functional insights into the various fractions of supraglacial microbial communities. Applying this framework directly in situ advances our ability to characterise dormancy in glacier ecosystems. Understanding the spectrum of metabolic states, and how they shift in response to rapid warming, is critical for predicting the resilience and ecological roles of glacier microbiomes in a fast-changing Arctic.

## Materials, methods, field report, and initial results

### Site description

**Figure 1.**
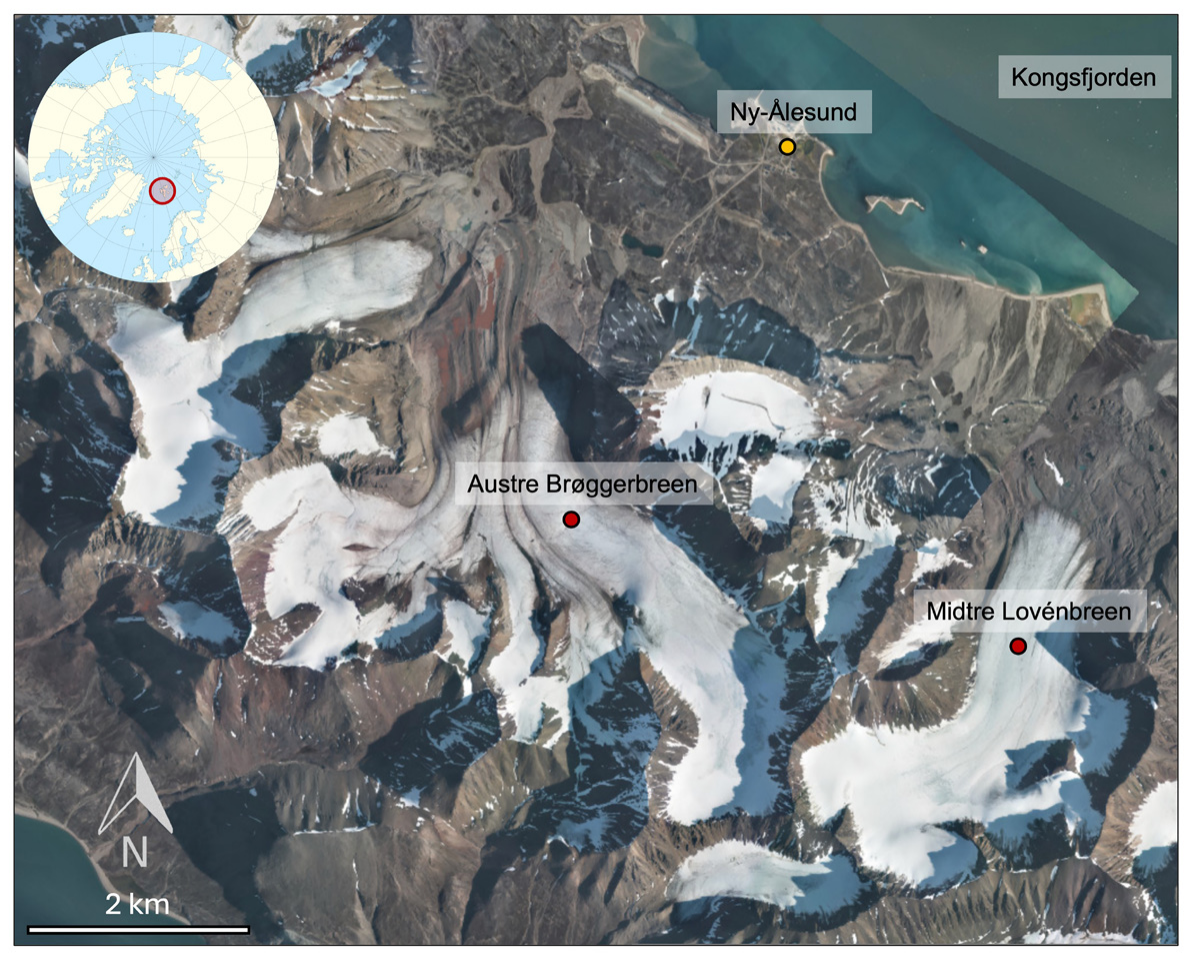
Map of the study sites. The location of the sampling sites are indicated by red pins, and the position of the Ny-Ålesund International Research Station ( https://nyalesundresearch.no/) is indicated by the yellow pin. The figure was created using an aerial photograph obtained from TopoSvalbard ( https://toposvalbard.npolar.no).

Ny-Ålesund, Svalbard, experiences a relatively mild climate for its high latitude (78.9°N), warmed by the West Spitsbergen Current. Ny-Ålesund has experienced an increase in mean annual air temperature of 3.9 °C since 1975 (data from Norwegian Polar Institute/Seklima, “Observations and Weather Statistics,” https://seklima.met.no/). Our study focussed on two retreating glaciers on northern Brøggeralvøya, located in north-west Spitsbergen: the polythermal Midtre Lovénbreen (78.88°N, 12.07°W) and cold-based Austre Brøggerbreen (78.88°N, 11.83°W). These glaciers are accessible by foot from Ny-Ålesund, located approximately 4.5 km and 3.0 km respectively (overland travel distance) from the settlement ( Figure 1). These sites have been chosen for sampling due to logistical convenience and because they have been widely studied over recent decades.

### Expedition team and logistics

The expedition team was composed of a group of interdisciplinary scientists with expertise spanning geomicrobiology, biochemistry, and field, experimental and modelling-based investigation. The field team was composed of four researchers from the Mediterranean Institute of Oceanography (Marseille, France): James A. Bradley (Expedition Leader and PI of the project), Rey Mourot (postdoctoral researcher in ecology and co-PI for the expedition), Eloi Martinez-Rabert (postdoctoral researcher in theoretical ecology and modelling), Sibylle Lebert (PhD student in molecular and environmental microbiology). The field team was supported by “on-shore” scientific collaborators including scientists at the Mediterranean Institute of Oceanography (Marseille, France) and external collaborators of the SIESTA project. The field expedition was co-financed by the European Research Council (ERC) under the European Union’s Horizon Europe Research and Innovation programme (Grant agreement No. 101115755, acronym SIESTA), and the French Polar Institute Paul-Émile Victor (IPEV) (Project 1308 - SIESTA), and hosted by the AWIPEV Arctic Research Base in Ny-Ålesund. The field science party was responsible for the sample collection, processing, field measurements, and overall project management and administration.

Reaching the sites involved travel by foot from Ny-Ålesund for approximately 3.5 to 5 km to reach the glacier snout, crossing small meltwater streams before ascending onto the glacier surface. On the ice, the terrain was relatively safe: melt channels, supraglacial lakes, and crevassed areas were clearly visible, as the expedition took place during the middle to late summer, when the glacier was mostly free of snow cover, and thus these features were easy to identify and avoid. Microspikes were worn over hiking boots in order to grip the ice.

During the campaign, air temperatures ranged between 3 to 12 °C off the ice, and 2 to 5 °C on the ice. Snow cover was absent from the tundra and largely absent from the glacier surfaces, except for residual snow in hollows and at higher elevations. All fieldwork was conducted under standard polar bear precaution protocols for Svalbard. The team was equipped with bolt-action rifles, flare pistols, radios, a satellite phone and an iridium GPS, and maintained constant awareness of surroundings at all times, with dedicated polar bear watch duties. Sampling and processing activities were carried out over eight consecutive days, without rest days, taking advantage of favourable weather conditions and in recognition that fieldwork opportunities in the Arctic may be rapidly compromised (through, for instance: polar bear encounters, illness, low visibility, unpredictable weather).

### Sampling methods

**Figure 2.**
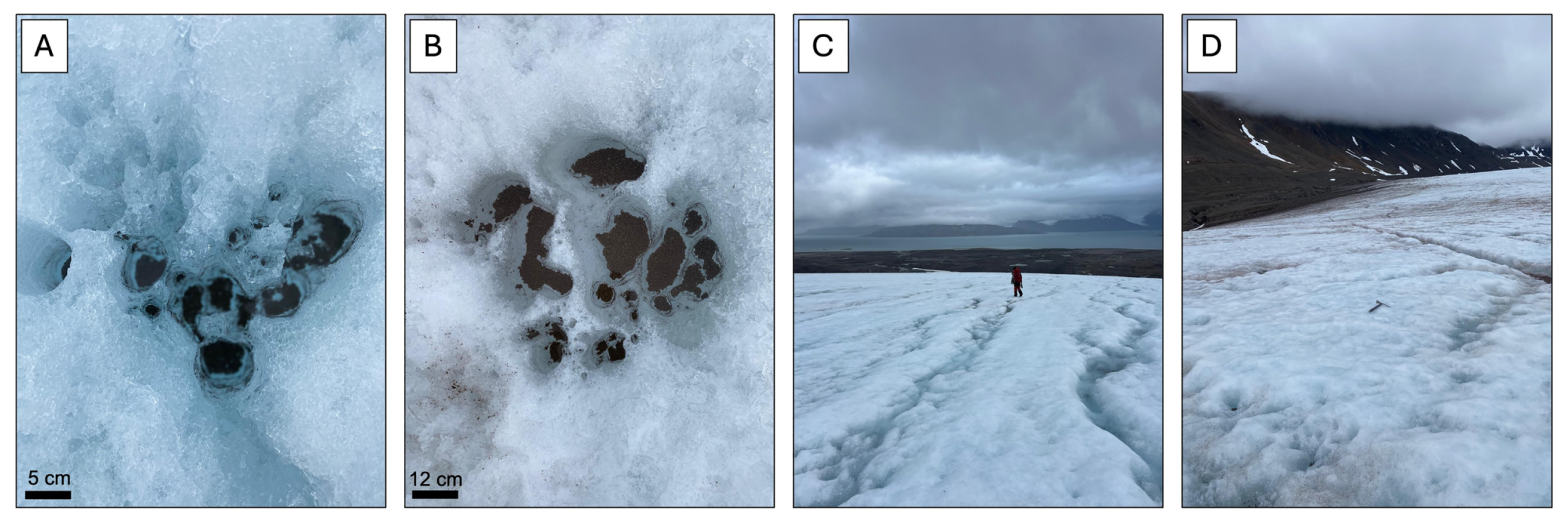
Photographs of the field site. **A**: Cryoconite holes on Midtre Lovénbreen; **B**: Cryoconite holes on Austre Brøggerbreen; **C**: The ice surface of Midtre Lovénbreen. A person is visible for scale; **D**: The ice surface of Austre Brøggerbreen. An ice axe is visible in the center of the photograph, for scale.

Samples were collected from both the ice surface habitat and cryoconite holes ( Figure 2). Ice surface material was collected by scraping the top two to five centimetres of the bare ice surface (i.e. the weathering crust) using ethanol-cleaned, field conditioned ice axes (with sterile gloves). The debris of the ice scrapings were homogenized in piles on the ice using ice axes, and placed into large sterile Whirl-Pak® bags. Cryoconite sediment and water from cryoconite holes were retrieved using sterile and field-conditioned turkey basters and transferred into small Whirl-Pak® bags and 50 mL Falcon tubes. In situ environmental measurements and observations were carried out alongside sampling, including visual assessments of surface conditions, temperature, and hydrological features, to provide contextual data for subsequent analyses and interpretation. Samples were transported back to the field laboratory in backpacks. This required consideration of weight, limited carrying capacity, and the fragility of Whirl-Pak® bags in contact with sharp ice fragments. Upon arrival, samples were aliquoted to be processed immediately or stored under controlled conditions until further analysis. A summary of the samples collected is presented in Table 1.

**Table 1.** A summary of the samples collected and the site characteristics.

Sample ID	Sample site	Habitat	Date collected	Latitude	Longitude	Altitude (m)	Air temperature (°C)	Water / ice temperature (°C)
S25.029	Midtre Lovénbreen	Cryoconite	19.07.2025	78°53.3321' N	12°02.8784' E	108	3.2	0.2
S25.030	Austre Brøggerbreen	Cryoconite	21.07.2025	78°53.6647' N	011°50.5816' E	117	4.6	0.3
S25.031	Austre Brøggerbreen	Ice surface	23.07.2025	78°53'39.86 N	011°50.3400' E	156	3.6	0.3
S25.032	Midtre Lovénbreen	Ice	25.07.2025	78°53'23.85 N	012°02.4782' E	142	2.8	0.2

### Logistical considerations to sampling and sample processing

Field sampling was immediately followed by laboratory work and intermittent rest periods. Each sample set consisted of at least 13 L of ice and 1 L of cryoconite water and sediment recovered from the glacier. While cryoconite samples could be processed immediately after being return to Ny-Ålesund, ice samples required melting prior to processing. To accelerate sample melting, sealed and double-bagged ice samples were immersed in warm water and removed just before all of the ice had melted. The ice remaining in sample bags maintained thermal equilibrium within the bag, and thus the sample temperature is maintained at close to 0°C throughout the melting process. This approach considerably reduced processing time and enabled rapid transition from field to laboratory analyses, thereby limiting microbial growth and other physiological changes during melting. We simultaneously prepared killed controls: autoclaving a portion of the melted sample twice. Departure from Ny-Ålesund in the morning and returning from the field in the evening led to late-night or early-morning processing. Team duties had to be allocated efficiently to ensure that all analyses could be carried out immediately on return from field sampling, to ensure the reliability of the results. Rest schedules for each member of the team were adapted so that sample processing, filtering, incubations, and oxygen measurements could occur simultaneously, whilst ensuring that field participants could be rested and alert for the next field excursion. 

### Protocol design

To capture a spectrum of microbial physiological states, we combined three complementary markers: EdU (DNA synthesis), BONCAT (de novo protein synthesis), and RSG (redox activity). Additionally, PMA was applied to a subset of samples to estimate the proportion of extracellular/free DNA and membrane-compromised cells ( Table 2). Samples were preserved in GlyTE to maintain cell integrity and ensure compatibility with downstream analyses (including flow cytometry, cell sorting, and sequencing). All steps of the protocol were rigorously tested and optimized at the Mediterranean Institute of Oceanography (Marseille, France), prior to the field campaign ( Table 3).

**Table 2.** A summary of physiological markers.

Marker	Activity measured	Principle	Incubation time (field)	Alternatives tested (lab)
EdU	DNA synthesis	Incorporation of a thymidine analogue during DNA replication.	6 h	-
BONCAT	Protein synthesis	Incorporation of AHA (methionine analogue) into newly synthesized proteins.	6 h	Incubation times of 6 h and 24 h
RSG	Redox activity	Reagent is reduced in metabolically active cells causing fluorescence.	20 min	Concentrations of 1 – 3 µL / mL sample and incubation times ranging from 15 to 45 minutes.
PMA	Membrane integrity / extracellular DNA	Penetrates compromised cells and binds to DNA, preventing PCR amplification.	1 h in the dark, followed by 15 min light exposure.	PI (incompatible with GlyTE).

**Table 3.** Overview of experimental steps applied to the samples. Each step is described with its purpose, principle, conditions, alternatives tested in the laboratory, and notes.

Step	Purpose	Principle	Conditions	Alternatives tested (lab)	Notes
Ice sample fast melting	Melt ice for incubation and filtering.	Double-bagged ice placed in warm water, removed from water just before all the ice melted completely.	Variable (15 – 60 min).	Passive melting at room temperature (too slow, risk of growth and activity change).	Maintains ~0°C until the final ice melts.
Filter rinsing	Remove markers from the sample before long-term preservation.	Filters rinsed with filtered MilliQ to remove the markers (HPG, EdU, RSG).	60 mL per filter.	Different concentrations of PBS for osmolarity + cell integrity assessment using fluorescence microscopy.	Ensures that no activity marking continues after the end of the incubation period.
Preservation (GlyTE)	Preserve cells at - 20 °C after incubation.	Samples fixed in GlyTE solution (glycerol, Tris, EDTA) for stability, FCM compatibility & DNA preservation.	Immediate after filter rinsing.	Formaldehyde; glutaraldehyde; direct freezing at - 20 °C.	Allows long-term storage, for later cell sorting and sequencing.

### Activity marking and sample processing

Subsamples were processed immediately upon return to the field laboratory, in order to capture the community state as close as possible to in situ conditions (T _0_) for downstream analyses.

***i) T _0_ Metagenomics and metatranscriptomics.*** For each sample, either 250 µL of cryoconite sediment or 500–1000 mL of melted ice (filtered) were transferred into Zymo DNA/RNA Miniprep tubes, with five replicates prepared per sample. Zymo DNA/RNA Shield was added following the manufacturer’s instructions, and samples were preserved at −20 °C until DNA/RNA extraction in the home laboratory.

***ii) BONCAT***. For ice samples, 10 replicate sterile culture flasks were filled with 500 mL fast-melted ice. Five flasks received 5 mL of HPG working solution (500 µM), while the remaining five served as non-labelled controls and received 5 mL of filtered MilliQ water instead. An additional flask was filled with 500 mL of autoclaved sample and 5 mL of HPG working solution to serve as a negative (killed) control.

For cryoconite, 2 mL of sediment–water mix was dispensed into 10 replicate 15 mL Falcon tubes. Five tubes received 200 µL of HPG working solution, and the other five tubes received 200 µL of filtered Milli-Q water, serving as non-labelled controls. One additional tube containing 2 mL of autoclaved sediment–water mix with 200 µL of HPG was prepared as a negative (killed) control.

All samples were incubated for six hours in the dark, in an ice bath. Following incubation, they were filtered onto 47 mm polycarbonate filters (0.22 µm). Filters were rinsed using 60 mL filtered MilliQ water to wash the marker from the filter and stop the labelling process. Three of the five non-labelled control filters were cut in half using a sterile scalpel: one half was transferred into a Zymo DNA/RNA Miniprep tube with 750 µL of Zymo DNA/RNA Shield for genomic analyses, and the other half was placed in a 2 mL cryotube for flow cytometry. These samples will be analyzed and compared to T _0_ metagenomic/metatranscriptomic samples (above) to assess potential bottle effects during incubation with BONCAT and EdU. The remaining filters were preserved in 2 mL cryotubes containing GlyTE for flow cytometry analyses. All tubes were stored and transported at −20 °C until further analysis.

***iii) EdU***. For ice samples, 10 replicate aliquots of 500 mL of melted ice were filtered onto 47 mm polycarbonate filters (0.22 µm). Each filter was placed into a 15 mL Falcon tube and covered with 2 mL of filtered Milli-Q water. Five tubes received 20 µL of EdU working solution, while the other five were incubated with 20 µL of filtered Milli-Q water as non-labelled controls. A negative (killed) control was prepared from 500 mL of autoclaved melted ice treated identically to the labelled samples.

For cryoconite samples, 2 mL of sediment–water mix was dispensed into 10 replicate 15 mL tubes. Five tubes received 20 µL of EdU working solution, while the remaining five received 20 µL of filtered Milli-Q water as non-labelled controls. An additional tube containing 2 mL of autoclaved sediment–water mix with 20 µL of EdU served as a negative (killed) control for the marker.

All tubes were gently vortexed at the start of incubation to ensure a homogeneous distribution of EdU. Samples were then incubated for six hours in the dark in an ice bath. Following incubation, samples were filtered onto 47 mm polycarbonate filters (0.22 µm). Filters were rinsed using 60 mL filtered MilliQ water to remove the marker from the filter and stop the labelling process. Filters were preserved in 2 mL cryotubes covered with GlyTE. All tubes were stored and transported frozen (at −20 °C) until further analysis.

***iv) RSG***. Due to the quantity and cost of reagent required for incubation (1 µL per 1 mL cryoconite (with ~10 ^6^ cells mL ^-1^ based on flow cytometry analyses of Midtre Lovénbreen cryoconite prior to the field campaign, data not shown), we adapted the RSG protocol for ice samples. To achieve sufficient cell concentrations for downstream analyses in reduced incubation volumes, ten replicate 500 mL aliquots of melted ice were filtered onto 47 mm polycarbonate filters (0.22 µm). Filters were placed into 15 mL Falcon tubes with 2 mL of Milli-Q water added to each tube. Five tubes received 20 µL of RSG working solution (0.1 mM), while the other five tubes were incubated with 20 µL of filtered Milli-Q water as non-labelled controls. A negative (killed) control was prepared using 500 mL of autoclaved melted ice treated identically to the labelled samples.

For cryoconite samples, 1 mL of cryoconite sediment-water mix was pipetted into ten replicate 2 mL tubes. 10 mL RSG working solution (0.1 mM) was added to five tubes, while the other five tubes received 10 µL filtered MilliQ-water as non-labelled controls. A negative (killed) control was prepared using 1 mL of autoclaved cryoconite treated identically to the labelled samples.

Each tube was gently vortexed at the start of incubation to ensure homogeneous distribution of RSG. All tubes were incubated for 20 minutes in the dark, in an ice bath in the fridge. Following incubation, samples were filtered onto 47 mm polycarbonate filters (0.22 µm) and then rinsed with filtered MilliQ water. Both filters (from the concentration and incubation steps) were placed together into the same 2 mL cryotube filled with GlyTE to ensure all cells were captured. Cryotubes were stored and transported at −20 °C until further analysis. Because of the short incubation time, we deemed it unnecessary to divide filters for genomic analyses for assessment of bottle effects during the incubation time,

***v) PMA***. For ice samples, five replicates of 300 mL of melted ice were filtered onto polycarbonate 0.22 uM, 47 mm filters. Each filter was cut in half using a sterile scalpel, with one half allocated for PMA treatment while the other half was used as control. 500 mL of filtered MilliQ water was then added to each tube.

For cryoconite samples, ten replicate 500 µL aliquots of cryoconite sediment were placed into 2 mL translucent tubes. Five randomly chosen tubes were labelled for PMA treatment (i.e. intracellular DNA [iDNA]), while the other five were used as controls (i.e. total DNA [tDNA]).

For PMA-treated tubes, 5 uL of PMA working solution (2 mM stock solution) was added to the tubes. For control tubes, 5 uL of MilliQ water was added. All cryoconite and ice samples were incubated in the dark, in an ice bath in the fridge for one hour, gently mixing the tubes every 15 minutes. Subsequently, the samples were placed on ice and exposed to a 500 W halogen lamp (at a distance of approximately 20 cm) for 15 min. After light exposure, the samples were frozen at -20°C and transported to the home-lab for analysis.

### Environmental data

Samples were also processed for dissolved inorganic nitrogen (NO _2_^−^, NO _3_^−^) and phosphate (PO _4_^3−^) analyses as follows: several millilitres of sample were flushed through the plastic syringe and single-unit 0.22 µM filter and discarded, to condition the plasticware. Each 60 mL polypropylene Nalgene vial was then rinsed three times with filtered sample, and then filled to three-quarters of its volume. Bottles were tightly capped and immediately frozen upright. Latex gloves were worn instead of nitrile, as the latter can release trace amounts of nitrogen- and sulfur-containing compounds into the samples, contaminating and interfering with sensitive measurements.

For trace-element analyses, samples were processed using the already-conditioned syringe, rinsing the 30 mL polypropylene Nalgene vials three times with filtered sample and discarding, before filling the vials to the top. Subsequently, 120 µL of HCl 37% was added to each vial to a final concentration of 0.4% v/v. Vials were then closed tightly and stored at 4 °C until analysis.

### Oxygen measurements

Oxygen measurements were conducted for a complementary assessment of microbial activity. Oxygen concentrations were measured within autoclaved 20 mL Presens sensor vials, via a polymer optical fiber using the Presens Fibox 4 Oxygen Meter. For cryoconite samples, two complementary approaches were tested: (i) pipetting a defined 2 mL volume of sediment into the vial, before filling up the remaining headspace of the vial with cryoconite water and closing tightly (ensuring no headspace), and (ii) filling up the vials with sediments to a consistent mark on the vial representing 10 ml (allowing time for sediments to settle and consolidate), and then filling the remaining headspace with cryoconite water, before capping. Both approaches presented advantages and limitations, particularly with respect to the amount of sediment required to obtain a clear signal of oxygen consumption, while still preserving sufficient material for parallel analyses. For ice samples, vials were filled with melted ice and tightly closed. Five vials were placed in ice baths in the dark in a fridge, and five vials were placed in ice baths outside in natural light. Bottles incubated in the dark captured oxygen consumption from community respiration alone, representing the metabolic demand of microbial assemblages in the absence of photosynthesis. Bottles incubated in the light captured both photosynthetic oxygen production and respiratory consumption. In addition to vials containing samples, a vial filled to the top with tap water, capped, and placed in each ice bath. This vial was opened at each measurement timepoint, in order to measure the temperature of the fluid (and thus the microcosms). This temperature was inputted into the FiBox4 prior to measurement. Oxygen measurements were conducted at time intervals of approximately 15 minutes during the first 3 hours. The timing of measurements was adjusted according to the magnitude of changes in O _2_ (which were rapid at first, and then slowed). We thus shifted to 1 to 2 hour intervals between measurements for the later stages of the incubations. It remains to be determined if screw-top vials provide a sufficient oxygen gas tight seal, or if crimp-sealed vials and butyl stoppers would be preferable. 

## Conclusions

The expedition resulted in the successful collection and processing of four samples across two glaciers (Midtre Lovénbreen and Austre Brøggerbreen) and two habitats (ice surface and cryoconite holes). These samples were processed for T _0_ amplicon, metagenomic and metatranscriptomic analyses, oxygen consumption measurements, geochemical composition, and cell-specific activity labelling assessments - which will later be further processed and analysed using flow cytometry, cell sorting, and genomic sequencing. Together, these datasets will provide a detailed picture of microbial communities and their physiological states inhabiting glacial ice and cryoconite during summer melting. These results will represent a valuable opportunity to characterize microbial functioning in situ in a remote High Arctic glacier environment, and to advance our understanding of the ecological roles of microbial dormancy in glacier surface habitats.

## Ethics and consent

Ethical approval and consent were not required.

## Data Availability

No new data were created or analysed in this study. Data sharing is not applicable to this article.

## References

[ref-1] Anesio AM Hodson AJ Fritz A : High microbial activity on glaciers: importance to the global carbon cycle. *Glob Chang Biol.* 2009;15(4):955–960. 10.1111/j.1365-2486.2008.01758.x

[ref-2] Anesio AM Lutz S Chrismas NAM : The microbiome of glaciers and ice sheets. *NPJ Biofilms Microbiomes.* 2017;3(1): 10. 10.1038/s41522-017-0019-0 28649411 PMC5460203

[ref-3] Bradley JA Trivedi CB Winkel M : Active and dormant microorganisms on glacier surfaces. *Geobiology.* 2023;21(2):244–261. 10.1111/gbi.12535 36450703 PMC10099831

[ref-4] Bradley JA : Microbial dormancy as an ecological and biogeochemical regulator on Earth. *Nat Commun.* 2025;16(1): 3909. 10.1038/s41467-025-59167-6 40280922 PMC12032139

[ref-5] Bradley JA Moncayo LM Gallo G : Svalbard winter warming is reaching melting point. *Nat Commun.* 2025;16(1): 6409. 10.1038/s41467-025-60926-8 40691432 PMC12280028

[ref-6] Couradeau E Sasse J Goudeau D : Probing the active fraction of soil microbiomes using BONCAT-FACS. *Nat Commun.* 2019;10(1): 2770. 10.1038/s41467-019-10542-0 31235780 PMC6591230

[ref-7] Edwards A Anesio AM Rassner SM : Possible interactions between bacterial diversity, microbial activity and supraglacial hydrology of cryoconite holes in Svalbard. *ISME J.* 2011;5(1):150–160. 10.1038/ismej.2010.100 20664552 PMC3105670

[ref-8] Fleming KM Dowdeswell JA Oerlemans J : Modelling the mass balance of northwest Spitsbergen glaciers and responses to climate change. *Ann Glaciol.* 1997;24:203–210. 10.3189/S0260305500012180

[ref-9] Hatzenpichler R Scheller S Tavormina PL : *In situ* visualization of newly synthesized proteins in environmental microbes using amino acid tagging and click chemistry. *Environ Microbiol.* 2014;16(8):2568–2590. 10.1111/1462-2920.12436 24571640 PMC4122687

[ref-10] Hodson A Anesio AM Tranter M : Ecol Monogr. *Ecological Monographs.* 2008;78(1):41–67. 10.1890/07-0187.1

[ref-11] Irvine-Fynn TDL Hanna E Barrand NE : Examination of a physically based, high-resolution, distributed Arctic temperature-index melt model, on Midtre Lovénbreen, Svalbard. *Hydrol Process.* 2014;28(1):134–149. 10.1002/hyp.9526

[ref-12] Jones SE Lennon JT : Dormancy contributes to the maintenance of microbial diversity. *Proc Natl Acad Sci U S A.* 2010;107(13):5881–5886. 10.1073/pnas.0912765107 20231463 PMC2851880

[ref-13] Kalyuzhnaya MG Lidstrom ME Chistoserdova L : Real-time detection of actively metabolizing microbes by redox sensing as applied to methylotroph populations in Lake Washington. *ISME J.* 2008;2(7):696–706. 10.1038/ismej.2008.32 18607374

[ref-14] Konopka MC Strovas TJ Ojala DS : Respiration response imaging for real-time detection of microbial function at the single-cell level. *Appl Environ Microbiol.* 2011;77(1):67–72. 10.1128/AEM.01166-10 21075887 PMC3019738

[ref-15] Kussell E Leibler S : Phenotypic diversity, population growth, and information in fluctuating environments. *Science.* 2005;309(5743):2075–2078. 10.1126/science.1114383 16123265

[ref-16] Lennon JT Jones SE : Microbial seed banks: the ecological and evolutionary implications of dormancy. * Nat Rev Microbiol.* 2011;9(2):119–130. 10.1038/nrmicro2504 21233850

[ref-17] Măgălie A Schwartz DA Lennon JT : Optimal dormancy strategies in fluctuating environments given delays in phenotypic switching. *J Theor Biol.* 2023;561: 111413. 10.1016/j.jtbi.2023.111413 36639023

[ref-18] Nocker A Cheung CY Camper AK : Comparison of propidium monoazide with ethidium monoazide for differentiation of live vs. dead bacteria by selective removal of DNA from dead cells. *J Microbiol Methods.* 2006;67(2):310–20. 10.1016/j.mimet.2006.04.015 16753236

[ref-19] Peoples LM Donaldson S Osuntokun O : Vertically distinct microbial communities in the Mariana and Kermadec trenches. *PLoS One.* 2018;13(4): e0195102. 10.1371/journal.pone.0195102 29621268 PMC5886532

[ref-20] Previdi M Smith KL Polvani LM : Arctic amplification of climate change: a review of underlying mechanisms. *Environ Res Lett.* 2021;16(9): 093003. 10.1088/1748-9326/ac1c29

[ref-21] Rantanen M Karpechko AY Lipponen A : The Arctic has warmed nearly four times faster than the globe since 1979. *Commun Earth Environ.* 2022;3(1): 168. 10.1038/s43247-022-00498-3

[ref-22] Salic A Mitchison TJ : A chemical method for fast and sensitive detection of DNA synthesis *in vivo*. *Proc Natl Acad Sci U S A* 2008;105(7):2415–2420. 10.1073/pnas.0712168105 18272492 PMC2268151

[ref-23] Shoemaker WR Lennon JT : Evolution with a seed bank: the population genetic consequences of microbial dormancy. *Evol Appl.* 2018;11(1):60–75. 10.1111/eva.12557 29302272 PMC5748526

[ref-24] Shoemaker WR Polezhaeva E Givens KB : Seed banks alter the molecular evolutionary dynamics of *Bacillus subtilis*. *Genetics.* 2022;221(2): iyac071. 10.1093/genetics/iyac071 35511143 PMC9157070

[ref-25] Zeng YX Yan M Yu Y : Diversity of bacteria in surface ice of Austre Lovénbreen glacier, Svalbard. *Arch Microbiol.* 2013;195(5):313–322. 10.1007/s00203-013-0880-z 23474777

