# Supplementary material for: SIESTA Project: Svalbard summer 2025 expedition report
Source: Open Res Eur. 2026 Jan 12;6:23. [Version 1] doi: 10.12688/openreseurope.21683.1 (PMC12902670; doi:10.12688/openreseurope.21683.1)
Supplement: Peer review history

# Reviewer report: Reviewer response for version 1

**DOI:** 10.21956/openreseurope.23456.r68833

**Published:** 2026-02-24

**Authors:** Siddarthan Venkatachalam

**Affiliations:** 1 National Centre for Polar and Ocean Research, Vasco da Gama, Goa, India

Congratulations to all the authors for producing this comprehensive expedition report, which was well-conceived and well-written to align with the project's main objectives. The report outlines sufficient details on the sampling plan, strategy, and protocol for each experiment, enabling anyone to replicate them in the future. I look forward to the project's outcome in the near future.

Are sufficient details of methods and materials provided to allow replication by others?

Yes

Is the rationale for creating the dataset(s) clearly described?

Yes

Are the datasets clearly presented in a useable and accessible format?

Partly

Are the protocols appropriate and is the work technically sound?

Yes

Reviewer Expertise:

Microbial Ecology and Genomics, Polar Biology

I confirm that I have read this submission and believe that I have an appropriate level of expertise to confirm that it is of an acceptable scientific standard.

# Reviewer report: Reviewer response for version 1

**DOI:** 10.21956/openreseurope.23456.r68829

**Published:** 2026-02-12

**Authors:** Mukan Ji

**Affiliations:** 1 Lanzhou University, Lanzhou, Gansu, China

Understanding the status of microorganisms is urgently needed to decipher the metabolic processes in glacier habitats. Previous studies on glacier microorganisms have relied heavily on amplicon sequencing and metagenome approaches, which are effective in providing an overview of the genetic novelty and metabolic capacities of entire microbial communities. However, these traditional methods fail to demonstrate the respective roles of individual microorganisms within the community. In the data note “SIESTA Project: Svalbard Summer 2025 Expedition Report,” Mourot et al. used four complementary approaches to differentiate the live and dormant members of the community. By combining these methods with amplicon and metagenomic sequencing, we can significantly enhance our understanding of the glacier microbiome. These protocols, covering sampling collection, pretreatment, and experimental procedures, are detailed and technically sound, making them useful for future researchers to follow. There is no data associated with this expedition report.

Are sufficient details of methods and materials provided to allow replication by others?

Yes

Is the rationale for creating the dataset(s) clearly described?

Yes

Are the datasets clearly presented in a useable and accessible format?

No

Are the protocols appropriate and is the work technically sound?

Yes

Reviewer Expertise:

Microbial community succession in glacier forefields

I confirm that I have read this submission and believe that I have an appropriate level of expertise to confirm that it is of an acceptable scientific standard.

# Reviewer report: Reviewer response for version 1

**DOI:** 10.21956/openreseurope.23456.r68834

**Published:** 2026-02-11

**Authors:** Mincheol Kim

**Affiliations:** 1 Korea Polar Research Institute, Incheon, Incheon, South Korea

The description of the SIESTA project is clear, and the manuscript is well written and straightforward.

 I have only a few minor comments below.

 The authors explained that several complementary methods were used to characterize a spectrum of microbial metabolic states rather than relying on a simply binary distinction (active vs inactive).  However, it is not sufficiently explained how each individual method, or their combined application, distinguishes among diffrent metabolic states.  Providing a more detailed description of how these methods collectively contributes to identifying the proposed metabolic spectrum would improve clarity and help readers better understand the research purpose.

 Regarding sample preservation, storage at -20C may be sufficient for DNA extraction. However, for RNA extraction followed by metatranscriptomic analyses, storage at temperatures below -60C is generally recommended to minimize RNA degradation and storage-related artifacts. This would help ensure that the measured transcriptomic profiles more accurately reflect in situ conditions at the time of sampling.

Are sufficient details of methods and materials provided to allow replication by others?

Yes

Is the rationale for creating the dataset(s) clearly described?

Yes

Are the datasets clearly presented in a useable and accessible format?

Yes

Are the protocols appropriate and is the work technically sound?

Yes

Reviewer Expertise:

Microbial ecology on polar regions

I confirm that I have read this submission and believe that I have an appropriate level of expertise to confirm that it is of an acceptable scientific standard.
